# Circulating miR-21 serves as a serum biomarker for hepatocellular carcinoma and correlated with distant metastasis

**DOI:** 10.18632/oncotarget.17211

**Published:** 2017-04-19

**Authors:** Xin Guo, Xiaohui Lv, Xing Lv, Yueyun Ma, Lin Chen, Yong Chen

**Affiliations:** ^1^ Department of General Surgery, Chinese PLA General Hospital, Beijing, P.R. China; ^2^ Department of Hepatobiliary Surgery, Xijing Hospital, Fourth Military Medical University, Xi’an, Shaanxi, P.R. China; ^3^ Department of Endoscopic Surgery, Chinese PLA 451st Hospital, Xi’an, Shaanxi, P.R. China; ^4^ Department of Gynecology and Obstetrics, Xijing Hospital, Fourth Military Medical University, Xi’an, Shaanxi, P.R. China; ^5^ Department of Clinical Laboratory Medicine, Xijing Hospital, Fourth Military Medical University, Xi’an, Shaanxi, P.R. China

**Keywords:** miR-21, AFP, hepatocellular carcinoma, ROC, biomarker

## Abstract

Serum miRNAs have gained great popularity to act as circulating biomarkers of several cancers. In this study, we aimed to evaluate the diagnostic efficiency of serum miR-21 as novel biomarkers for patients with hepatocellular carcinoma (HCC) and other controls. A total of 533 individuals were recruited and conducted in a two-step analysis. The pilot group included 40 HCC patients and 40 healthy donors. The expression levels of miR-21 were significantly higher in primary HCC tissues than in adjacent noncancerous tissues (P<0.0001). HCC patients exhibited significantly higher serum levels of miR-21 than HD (P<0.0001). In the verification group, the mean serum levels of miR-21 in 175 patients with HCC were significantly higher than in 64 with CHB, 78 with LC and 136 HD (all P<0.0001). ROC curves demonstrated that the AUC of miR-21 was 0.849, sensitivity 82.1% and specificity 83.9%. Furthermore, serum miR-21 maintained its diagnostic efficiency in AFP-negative HCC subgroups with AUC 0.831, sensitivity 81.2% and specificity 83.2%. The serum levels of miR-21 could distinguish HCC from CHB and LC (AUC 0.789, sensitivity 76.9%, specificity 85.7% and AUC 0.814, sensitivity 80.8%, specificity 72.9%, respectively). In addition, the serum levels of miR-21 were significantly associated with clinical stage (P=0.006) and distant metastasis (P=0.000). Thus, our findings suggest that miR-21 together with AFP may help enhance the diagnosis of HCC, especially of AFP-negative HCC, and could distinguish HCC from CHB and LC.

## INTRODUCTION

Hepatocellular carcinoma (HCC) is the second most common gastrointestinal solid tumors and remains the second leading cause of cancer-related death in China [1]. It is estimated that more than half of newly diagnosed cases of HCC and cancer-related deaths may occur in China [2]. Although surgical resection remains the potential curative treatment for patients with HCC, only 30-40% ones are operable partly due to the lack of effective methods of diagnosis in time. A-fetoprotein (AFP) is the only biomarker commonly used for screening of HCC. However, the diagnostic efficiency of α-fetoprotein remains unsatisfied for its elevations in patients with liver benign diseases (liver cirrhosis and chronic hepatitis) and screening of early-stage HCC [3]. Thus, identification of novel biomarkers for HCC to complement AFP is urgently needed.

MicroRNAs (miRNAs) are receiving increasing attentions and emerging as regulatory molecules in development and progression of various cancers [4, 5]. Many miRNAs have been identified to be dysregulated in cancers and serve as ideal biomarker candidates for their stable expression and resistance to endogenous RNase [6, 7]. As shown by many studies, the unique serum miRNAs expression profiles may shed new lights on cancer diagnosis including HCC [8–10].

MiR-21, located on the chromosome of 17q23.2, has been found to be dysregulated in several cancers including lung cancer [11, 12], breast cancer [13, 14] and pancreatic cancer [15, 16]. The high levels of miR-21 expression in cancers have been reported to be correlated with tumor proliferation, invasion and metastasis [17–20]. Huang and colleagues indicated that miR-21 was deregulated in HCC tissues and suggested its prognostic value for HCC [21]. Wang and colleagues noted that HCC patients had higher serum levels of miR-21 than healthy controls [22]. Wang and colleagues found that the serum levels of miR-21 reflected the HCC stages and pathological changes in a rat model [23]. However, all these studies had limitations including small sample size, lack of controls with liver benign diseases and simply measurement at tissue levels rather than serum levels. Thus, we performed a large-cohort, single-center investigation to evaluate the diagnostic efficiency of miR-21 for HCC.

## RESULTS

### Study design

Several studies have indicated that miR-21 was frequently over-expressed and played an oncogenic role in various cancers, including HCC. Thus, we performed a two-step analysis to evaluate the diagnostic efficiency of miR-21 for patients with HCC and controls. First, we confirmed the high expression levels of miR-21 in HCC tissue samples and corresponding serum samples in pilot group. Then, we further confirmed the diagnostic efficiency of miR-21 by large-scale cohorts with more control groups in verification group. We recruited 533 individuals, 80 in the pilot group and 453 in the verification group. The clinical characteristics of patients with HCC and control groups in pilot group and verification group are presented in Table 1.

**Table 1:** Clinical characteristics of individuals pilot group and verification group

Characteristics	Pilot Group (n=80)		Verification group (n=453)			
	HCC	HD	HCC	CHB	LC	HD
No. of patients	40	40	175	64	78	136
Gender (male/female)	25/15	27/13	97/61	39/25	47/31	75/61
Age (years)Mean ± SEM	56.2 ± 4.5	52.6 ± 5.1	54.3 ± 5.5	43.4 ± 3.7	52.3 ± 5.7	51.7 ± 6.3
AFP (ng/ml)Mean ± SEM	346.7 ± 41.3	13.7 ± 1.3	402.6 ± 24.1	246.7 ± 26.4	94.7 ± 25.3	14.0 ± 0.8
Tumor size (mm)Mean ± SEM	4.7 ± 2.1	NA	4.9 ± 2.4	NA	NA	NA
Pathological differentiation (well/moderate/poor)	8/19/13	NA	35/78/62	NA	NA	NA
Clinical stage (I/II/III/IV)	5/19/14/2	NA	33/74/56/12	NA	NA	NA

HCC: hepatocellular carcinoma; CHB: chronic hepatitis B infection; LC: liver cirrhosis; HD: healthy donors; SEM: standard error of the mean; NA: not available.

### The general diagnostic efficiency of miR-21 and AFP

**Figure 1:**
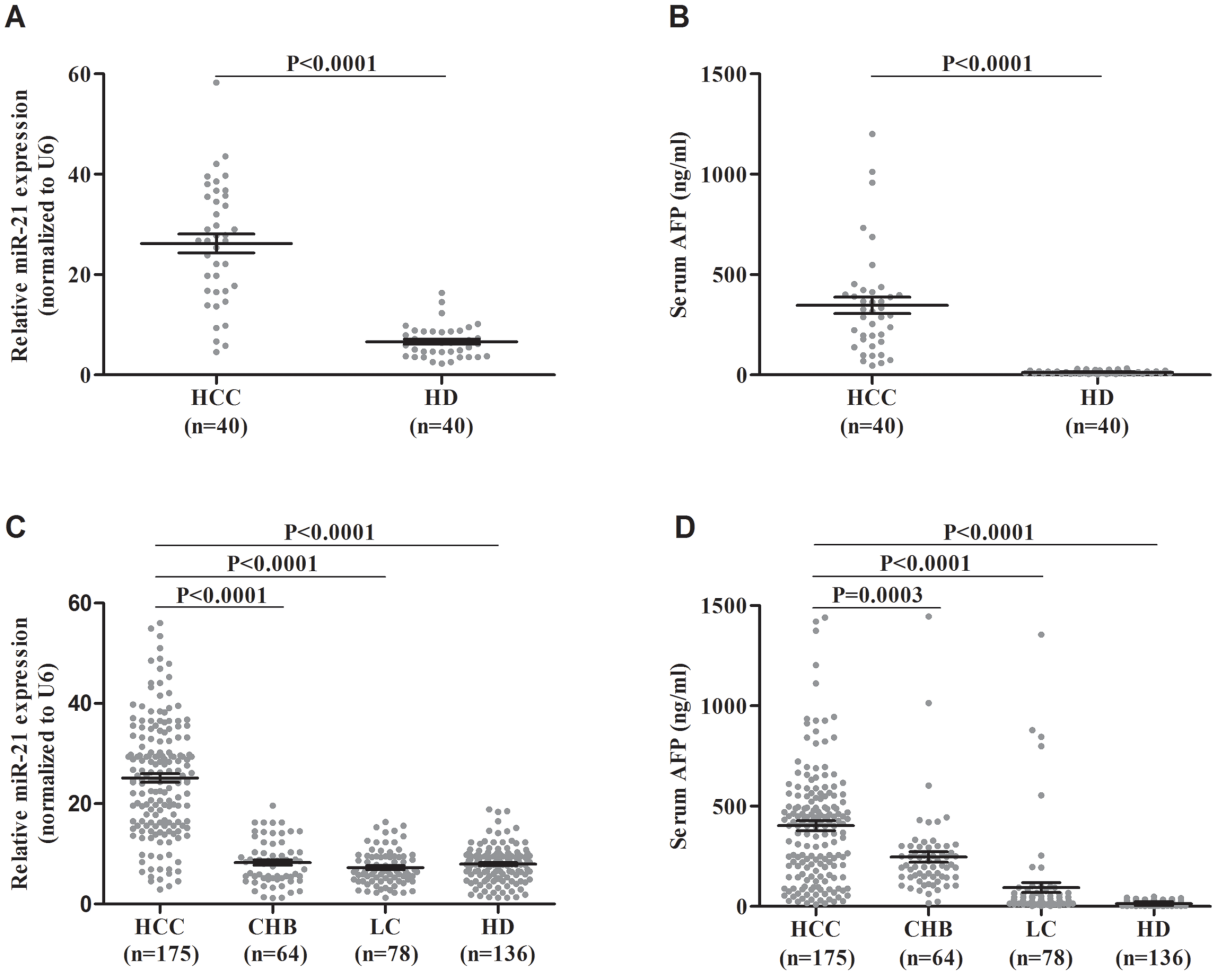
Results for serum miR-21 and AFP in the diagnosis of HCC **(A)** miR-21 for pilot group. **(B)** AFP for pilot group. **(C)** miR-21 for verification group. **(D)** AFP for verification group. Black horizontal lines are means, and error bars are SEMs. HCC: hepatocellular carcinoma; CHB: chronic hepatitis B virus infection; LC: liver cirrhosis; HD: healthy donor; SEM: standard error of the mean.

**Figure 2:**
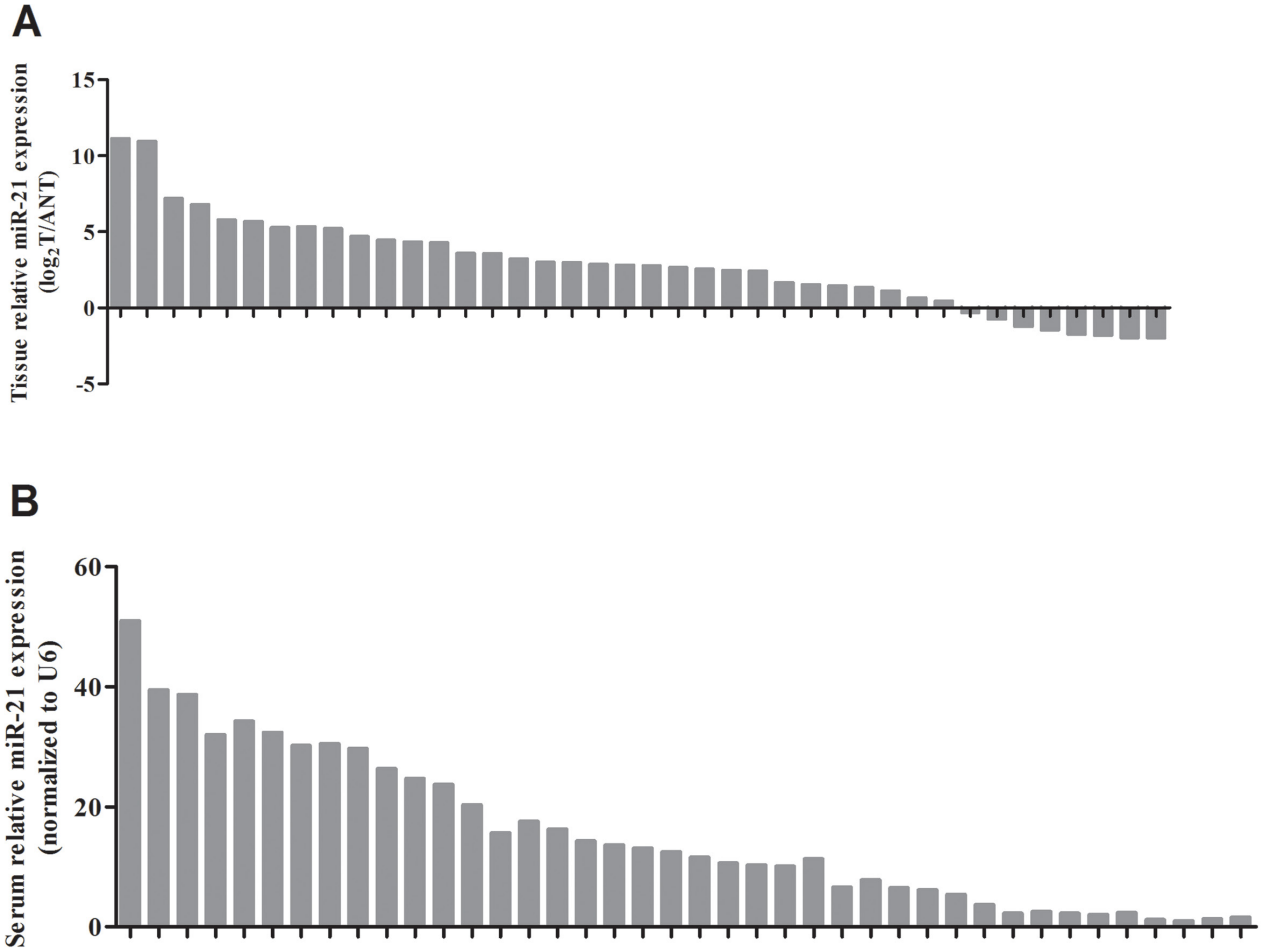
Parallel expressions of miR-21 in HCC tissue and serum samples **(A)** miR-21 levels for HCC tissue samples. **(B)** miR-21 levels for HCC serum samples. HCC: hepatocellular carcinoma.

**Figure 3:**
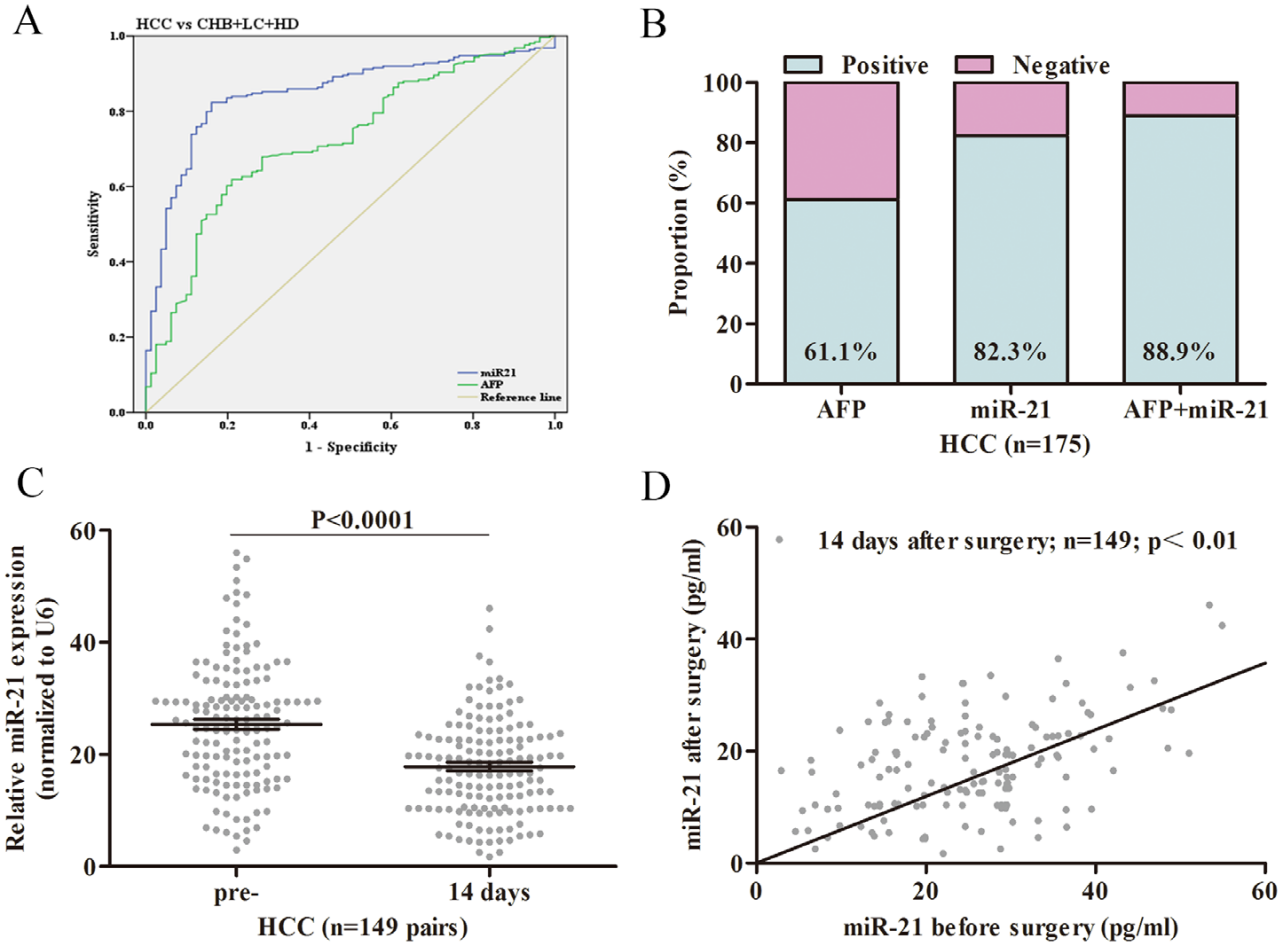
The general diagnostic efficiency of miR-21 and AFP for HCC **(A)** ROC curves of miR-21 and AFP for patients with HCC versus three controls. **(B)** The positive rates of miR-21, AFP or both in patients with HCC. **(C)** miR-21 levels in HCC patients pre- and 14 days after surgery and healthy donors. **(D)** Scatter plot of miR-21 levels in serum samples preoperative and 14 days after surgery. ROC: receiver operating characteristic; HCC: hepatocellular carcinoma; CHB: chronic hepatitis B virus infection; LC: liver cirrhosis; HD: healthy donor; pre-: preoperative.

Serum levels of miR-21 were significantly higher in patients with HCC in pilot group than in healthy donors (26.20±1.89 vs. 6.65±0.50 folds, P<0.0001, Figure 1A). As expected, serum levels of AFP were also increased in patients with HCC in pilot group than in healthy donors (346.70±41.28 vs. 13.74±1.31 ng/ml, P<0.0001, Figure 1B). Moreover, the expression levels of miR-21 in primary HCC tissue samples were up-regulated than adjacent noncancerous tissue samples (Figure 2A) and serum levels were mostly in accordance with corresponding tissue levels (35 of 40 cases, Figure 2B). Next, we further assessed the serum levels of miR-21 and AFP in verification group. The serum levels of miR-21 were dramatically higher in HCC in comparison with CHB (25.11±0.85 vs. 8.29±0.54 folds, P<0.0001), LC (25.11±0.85 vs. 7.26±0.40 folds, P<0.0001) and HD (25.11±0.85 vs. 7.95±0.30 folds, P<0.0001, Figure 1C). The three control groups showed no statistical significance. The serum levels of AFP were higher in patients with HCC in verification group than in healthy donors (402.60±24.09 vs. 14.01±0.84 ng/ml, P<0.0001), however, significant differences were also detected between CHB, LC and HD (246.70±26.42 vs. 14.01±0.84 ng/ml, P<0.0001; 94.65±25.29 vs. 14.01±0.84 ng/ml, P<0.0001, respectively, Figure 1D). Moreover, the serum miR-21 levels of 14 days after surgery in patients with HCC were significantly decreased compared with preoperative ones (25.36±0.90 vs. 17.85±0.78 folds, n=149 pairs, P<0.0001, Figure 3C and 3D). These results of pilot group and verification group preliminarily indicated the significant association of HCC and the miR-21 levels, and the high false positivity of AFP in distinguishing HCC, CHB and LC.

Then, we constructed ROC curves to assess the diagnostic efficiency of miR-21 compared with AFP for HCC. The optimal diagnostic cutoff for miR-21 (AUC 0.849, 95% CI 0.803–0.894, sensitivity 82.1%, specificity 83.9%) was 8.023 fold, and AFP (AUC 0.722, 95% CI 0.661–0.783, sensitivity 68.7%, specificity 62.5%, Figure 3A) was 16.42 ng/ml. In addition, although the positive rate of miR-21 for HCC patients was higher than AFP, there was no statistical difference (82.3% vs. 61.1%, P=0.0821, Figure 3B). Thus, serum levels of miR-21 exhibited better performance than AFP in diagnosing HCC with CHB, LC and HD.

### The diagnostic efficiency of miR-21 in discriminating HCC, LC and CHB

**Figure 4:**
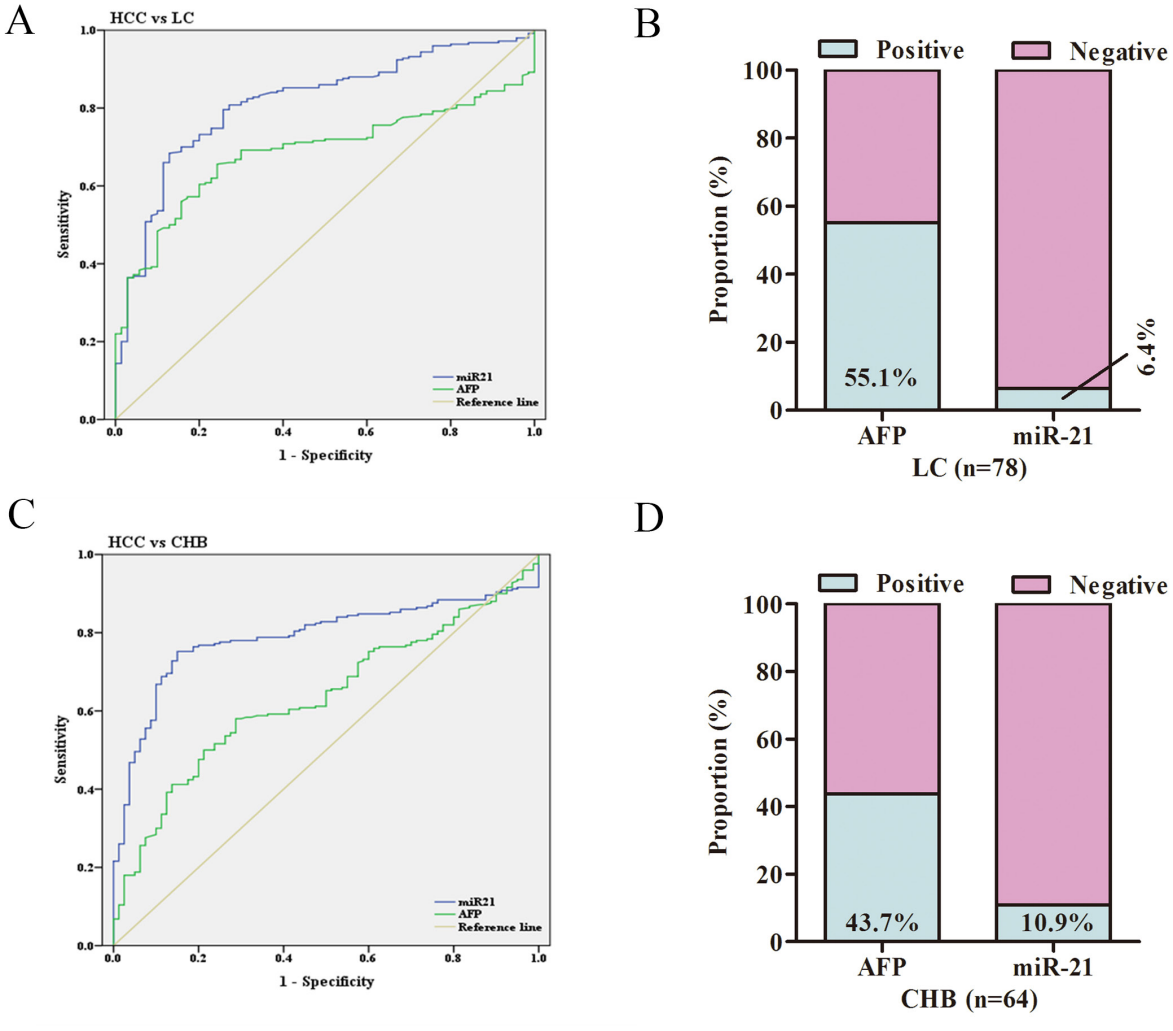
The diagnostic efficiency of miR-21 in subgroups of HCC **(A)** ROC curves of miR-21 and AFP for patients with HCC versus LC. **(B)** The positive rates of miR-21 and AFP in patients with LC. **(C)** ROC curves of miR-21 and AFP for patients with HCC versus CHB. **(D)** The positive rates of miR-21 and AFP in patients with CHB. HCC: hepatocellular carcinoma; CHB: chronic hepatitis B virus infection; LC: liver cirrhosis; HD: healthy donor; ROC: receiver operating characteristic.

It is often criticized for AFP in discriminating patients with HCC and LC by increased false positivity. In this study, we further compared miR-21 and AFP in HCC and LC groups. The ROC curves showed that miR-21 had greater performance than AFP (AUC 0.814, 95% CI 0.761–0.867, sensitivity 80.8%, specificity 72.9% versus AUC 0.686, 95% CI 0.628–0.744, sensitivity 70.4%, specificity 71.5%, Figure 4A). In addition, the positive rate of miR-21 was 6.4% in LC group, whereas serum AFP was with a high positive rate of 55.1% (Figure 4B). In terms of the risk group of HCC (CHB), miR-21 also had a better performance than AFP (AUC 0.789, 95% CI 0.739–0.839, sensitivity 76.9%, specificity 85.7% versus AUC 0.634, 95% CI 0.571–0.697, sensitivity 59.3%, specificity 69.7%, Figure 4C). The proportion of patients with CHB were detected with higher AFP positive rate than miR-21 (43.7% vs. 10.9%, Figure 4D). These results suggested that miR-21 exhibited a greater diagnostic performance in discriminating HCC, LC and CHB than AFP.

### The diagnostic efficiency of miR-21 in AFP-negative HCC

**Figure 5:**
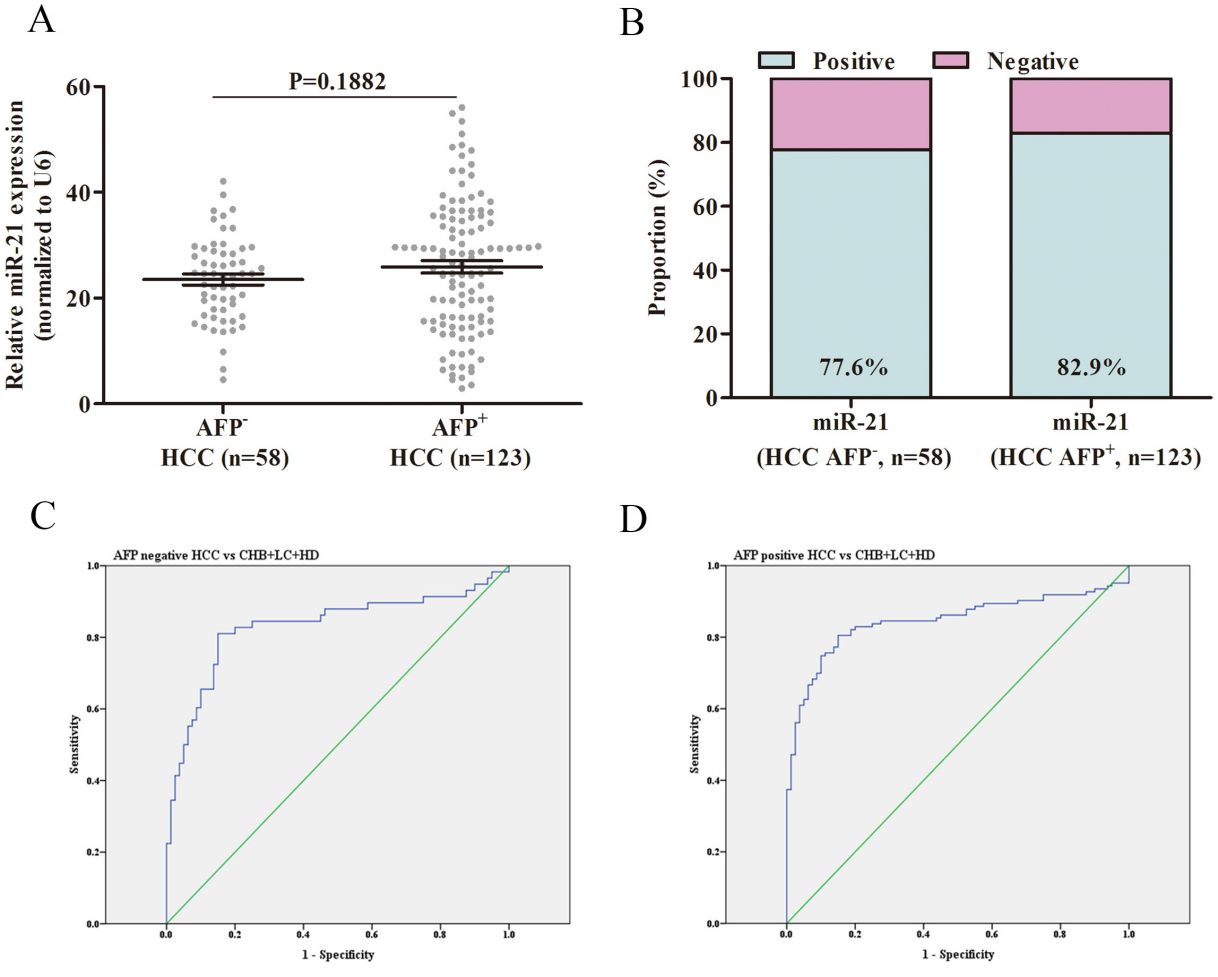
The diagnostic efficiency of miR-21 in AFP-negative HCC **(A)** miR-21 levels in AFP-negative and AFP-positive HCC groups. **(B)** The positive rates of miR-21 in AFP-negative and AFP-positive HCC groups. **(C)** ROC curves of miR-21 for patients with AFP-negative HCC versus three controls. **(D)** ROC curves of miR-21 for patients with AFP-positive HCC versus three controls. HCC: hepatocellular carcinoma; CHB: chronic hepatitis B virus infection; LC: liver cirrhosis; HD: healthy donor; ROC: receiver operating characteristic.

AFP has been widely used for diagnosing and monitoring treatment response for patients with HCC. However, it has been identified that 30-40% of HCC patients are of AFP-negative status, limiting the use of AFP. In this study, the serum levels of miR-21 showed no significance in AFP-positive and AFP-negative HCC subgroups (23.52±1.04 vs. 25.90±1.15 folds, P<0.0001, Figure 5A). For AFP-negative subgroups, ROC curves showed that miR-21 had a great performance (AUC 0.831, 95% CI 0.756–0.905, Figure 5C) with a sensitivity of 81.2% and a specificity of 83.2%). For AFP-positive subgroups, ROC curves indicated that miR-21 remained its great performance (AUC 0.846, 95% CI 0.790-0.901, Figure 5D) with a sensitivity of 80.3% and a specificity of 82.9%. Additionally, serum levels of miR-21 were assessed with a positive rate of 77.6% (45/58) in AFP-negative HCC group, and were with a positive rate of 82.9% (102/123, Figure 5B) in AFP-positive HCC group. The above results suggested that miR-21 remains its diagnostic efficiency in AFP-negative HCC subgroups. All the results of ROC curves were presented in Supplementary Table 1.

### Correlations between miR-21 and clinical characteristics of HCC

We evaluated the associations of serum levels of miR-21 and clinicopathologic characteristics of patients with HCC. As presented in Supplementary Table 2, the serum levels of miR-21 were significantly correlated with HCC clinical stage and distant metastasis (P=0.006 and P=0.000, respectively). The positive rate of miR-21 in HCC patients with distant metastasis was 83.3% (10/12), indicating that serum levels of miR-21 may be correlated with distant metastasis.

## DISCUSSION

Cancer-specific alterations of miRNAs expression are common in various cancers and act as critical roles in cancer progression. More and more evidence indicated that circulating serum miRNAs served as promising biomarkers for diagnosing and monitoring treatment response [24]. Circulating miRNAs have been found to be stable in serum, suggesting its potential ability to be diagnostic biomarkers [25]. Thus, identification of circulating miRNAs biomarkers may not only serve as a novel diagnostic tool, but also shed new lights to identify new HCC therapy targets.

HCC is a common digestive system malignancy with high mortality rate [1]. Currently, the diagnosis of HCC mainly defined by combination of imaging examinations and serum AFP levels. However, AFP is often criticized for its high false positivity in distinguishing HCC and LC. In addition, it is estimated that 30-40% of all HCC patients are of AFP-negative status, making it difficult to diagnose and assess treatment response [2]. Thus, identifying a new biomarker that could complement AFP is of great importance. For now, several miRNAs have been identified as novel biomarkers for HCC. Chen et al found that miRNA-221 exhibited high levels in HCC tissue and patients with high miRNA-221 had a shorter survival time [26]. Xiang et al identified that serum miRNA-34 levels can predict bone metastasis in HCC patients [27]. Ji et al found that serum miRNA-15b levels predict poor prognosis for patients with HCC after surgery [28].

In this study, we conducted a two-step analysis to evaluate whether serum miR-21 had the potential ability to be a new biomarker for detecting HCC. In pilot group, we assessed the expression of miR-21 in HCC tissue samples and adjacent noncancerous tissue samples, and confirmed the high miR-21 expression in primary HCC tissue samples, which were consistent with previous reports [21]. Meanwhile, we identified that healthy donors exhibited lower serum levels of miR-21 than HCC. Taken together, these findings suggested that miR-21 exhibited a potential ability to diagnose HCC compared with healthy individuals. In verification group, we discovered that postoperative serum levels of miR-21 were decreased than preoperative serum samples, suggesting that serum miR-21 levels may be correlated with tumor burden, namely, HCC cells are likely to be the origin of serum miR-21. When patients with CHB and LC were included as controls, miR-21 exhibited a better diagnostic efficiency than AFP for patients with HCC, especially for AFP-negative HCC patients. In subgroups of HCC, miR-21 remained its diagnostic efficiency in distinguishing HCC from CHB and LC. All the above results indicate that miR-21 could serve as a novel serum biomarker for HCC and has the potential ability to assess tumor burden and treatment response. However, large-scale cohorts and strict study design are needed to further confirm the diagnostic role of miR-21.

There were several limitations in our study. First, this study is single-center and retrospective in nature, so there is likely selection bias and limitation of the generalizability of these findings. Second, the number of patients is still not large enough, and several other digestive system cancers should be also included as controls. Thus, we will plan to perform a prospective, large-scale and multiple-center study to further clarify the diagnostic role of miR-21 for HCC, and this study is currently in preparation. However, to our knowledge, this study was the first one to conduct a two-step analysis for the diagnostic evaluation of miR-21 for patients with HCC.

In summary, our findings suggest that serum miR-21 levels may help enhance the diagnosis of HCC, especially for AFP-negative HCC, and could distinguish HCC from CHB and LC.

## MATERIALS AND METHODS

### Study population

Serum specimens were obtained from the Department of clinical laboratory medicine, Xijing Hospital, the Fourth Military Medical University. 175 patients with primary HCC, 64 patients with chronic HBV infection (CHB), 78 patients with liver cirrhosis (LC) and 136 healthy donors (HD) were enrolled from August 2013 to April 2015. All the serum samples were collected before patients received any treatment, such as surgery, chemotherapy or radiotherapy, prior to the investigational surgery. Primary cancer tissues were verified by pathologists. Histological classification and clinical staging of HCC were performed according to the Barcelona Clinic Liver Cancer (BCLC) staging system. Chronic hepatitis B virus infection was defined based on continuous DNA quantitation higher than 10^3^ copies/ml and elevated levels of alanine aminotransferase. Diagnosis of liver cirrhosis included liver bioscopy, clinical manifestation of ascites, portal hypertension, splenomegaly, and laboratory tests. The healthy donors were enrolled for no abnormalities of blood tests, abdominal ultrasound and other malignant cancers. The venous blood samples were standing at room temperature for 45 min before centrifuging at 1000 rpm for 30 min. The supernatant serum was transferred and stored at -70°C until assay. This study was approved by the Ethics Committee of Xijing Hospital, the Fourth Military Medical University.

### Tests of miR-21-RNA extraction and qRT-PCR

Serum total RNA was isolated with miRNAs Serum Kit (Qiagen, Germany) strictly following the manufacturer's descriptions. RNA concentrations were measured by assessing the absorbance at 260 nm. Quantitative RT-PCR was performed as described in the manual instructions (Qiagen, Germany). The primers of miR-21 were purchased from Qiagen (MS00013216). U6 was chosen and calculated as an internal control. miR-21 levels were normalized to U6 and melt curves were performed to analyze the specificity. The results for each serum sample were presented with the 2^-ΔΔCt^ method as previously described [29]. Briefly, every single sample was calculated by the differences of threshold cycle (CT) between miR-21 and U6: Δ_CT_= (CT_ΔmiR-21_–CT_ΔU6_).

### Tests of AFP-ELISA

The serum levels of AFP were measured by commercially available kit (Abcam, UK) following the manufacturer's recommendations. Briefly, 50 μl of each serum samples were added into the plates (precoated with rabbit anti-human monoclonal antibody) and incubated for 1 h at room temperature. After washing with buffers for three times, 50 μl second antibody conjugated with HRP (mouse anti-rabbit polyclonal antibody) was loaded into each plate and incubated for 45 min. Once again, after washing with buffers for 3 times, the plates were incubated with HRP solution for 30 min and then with color agents for 10 min in the dark. Stopping buffer was incubated to terminate the reaction. AFP concentrations were shown by detecting absorbance at 450 nm.

### Statistical analysis

Analysis was performed with Prism 5 software (GraphPad) and SPSS 18.0 to assess between-group differences with Student's t test. ROC curves were calculated to assess the diagnostic efficiency of serum miR-21 levels and AFP levels. The AUC was used to measure the predictive value. Two-sides P values of < 0.05 were considered with a statistical significance.

## SUPPLEMENTARY MATERIALS TABLES



## Abbreviations

HCC: hepatocellular carcinoma
CHB: chronic hepatitis B virus infection
LC: liver cirrhosis
HD: healthy donors
ROC: receiver operating characteristics
AUC: area under curve
AFP: α-fetoprotein
ELISA: enzyme-linked immunosorbent assay
HRP: horse radish peroxidase
