# Supplementary material for: Circulating miR-21 serves as a serum biomarker for hepatocellular carcinoma and correlated with distant metastasis
Source: Oncotarget. 2017 Apr 19;8(27):44050–8. doi: 10.18632/oncotarget.17211 (PMC5546461; doi:10.18632/oncotarget.17211)
Supplement: Supplementary file 1 [file oncotarget-08-44050-s001.pdf]

Circulating miR-21 serves as a serum biomarker for hepatocellular carcinoma and correlated with distant metastasis

SUPPLEMENTARY TABLES

Supplementary Table 1: Results of ROC curves for serum miR-21 and AFP in the diagnosis of HCC and controls

Variables	AUC	95%CI	Sensitivity	Specificity
**HCC vs CHB, LC and HD**				
miR-21	0.849	0.803-0.894	82.1%	83.9%
AFP	0.722	0.661-0.783	68.7%	62.5%
**HCC vs LC**				
miR-21	0.814	0.761-0.867	80.8%	72.9%
AFP	0.686	0.628-0.744	70.4%	71.5%
**HCC vs CHB**				
miR-21	0.789	0.739-0.839	76.9%	85.7%
AFP	0.634	0.571-0.697	59.3%	69.7%
**AFP negative HCC vs CHB+LC+HD**				
miR-21	0.831	0.756-0.905	81.2%	83.2%
**AFP positive HCC vs CHB+LC+HD**				
miR-21	0.846	0.790-0.901	80.3%	82.9%

ROC: Receiver Operating Characteristic; AUC: area under curve; CI: confidence interval; HCC: hepatocellular carcinoma; CHB: chronic hepatitis B infection; LC: liver cirrhosis; HD: healthy donors; AFP: α-fetoprotein.

Supplementary Table 2: Correlation between miR-21 levels and clinical characteristics in HCC patients in verification group

Characteristic	No. (n=175)	Serum miR-21 levels		*P* value
		High (n=107)	Low (n=68)	
Gender				0.598
Male	97	61 (57.0%)	36 (52.9%)	
Female	78	46 (43.0%)	32 (47.1%)	
Age (years)				0.336
<50	77	44 (41.1%)	33 (48.5%)	
≥50	98	63 (58.9%)	35 (51.5%)	
Tumor size (cm)				0.516
<5	108	64 (59.8%)	44 (64.7%)	
≥5	67	43 (40.2%)	24 (35.3%)	
Tumor number				0.076
Solitary	97	65 (60.7%)	32 (47.1%)	
Multiple	78	42 (39.3%)	36 (52.9%)	
Serum AFP levels (ng/ml)				0.254
<400	117	75 (70.1%)	42 (61.8%)	
≥400	58	32 (29.9%)	26 (38.2%)	
Pathological differentiation				0.582
Well+Moderate	115	72 (67.3%)	43 (63.2%)	
Poor	60	35 (32.7%)	25 (36.8%)	
Clinical stage				**0.006***
I+II	107	74 (69.2%)	33 (48.5%)	
III+IV	68	33 (30.8%)	35 (51.5%)	
Distant metastasis				**0.000***
Absent	163	97 (98.1%)	66 (85.3%)	
Present	12	10 (1.9%)	2 (14.7%)	

*Statistically significant. The extent of miR-21 serum levels was based on cutoff values defined by ROC curves. High level of serum miR-21 was defined as above the cutoff value, and low level of serum miR-21 was defined as below the cutoff value.
